# The Effect of High-Dose Insulin Analog Initiation Therapy on Lipid Peroxidation Products and Oxidative Stress Markers in Type 2 Diabetic Patients

**DOI:** 10.1155/2013/513742

**Published:** 2013-03-17

**Authors:** Hazal Tuzcu, Ibrahim Aslan, Mutay Aslan

**Affiliations:** ^1^Department of Medical Biochemistry, Akdeniz University Medical School, 07070 Antalya, Turkey; ^2^Endocrinology Clinic, Antalya Research and Education Hospital, 07100 Antalya, Turkey

## Abstract

Effect of high-dose insulin analog initiation therapy was evaluated on lipid peroxidation and oxidative stress markers in type 2 diabetes mellitus (T2DM). Twenty-four T2DM patients with HbA1c levels above 10% despite ongoing therapy with sulphonylurea and metformin were selected. Former treatment regimen was continued for the first day followed by substitution of sulphonylurea therapy with different insulin analogs. Glycemic profiles were determined over 72 hours by Continuous Glucose Monitoring System (CGMS), and blood/urine samples were collected at 24 and 72 hours. Insulin analog plus metformin treatment significantly reduced glucose variability. Plasma and urine lipid peroxidation were markedly decreased following insulin analog plus metformin treatment. No correlation existed between glucose variability and levels of plasma and urine oxidative stress markers. Likewise, changes in mean blood glucose from baseline to end point showed no significant correlation with changes in markers of oxidative stress. On the contrary, decreased levels of oxidative stress markers following treatment with insulin analogs were significantly correlated with mean blood glucose levels. In conclusion, insulin plus metformin resulted in a significant reduction in oxidative stress markers compared with oral hypoglycemic agents alone. Data from this study suggests that insulin analogs irrespective of changes in blood glucose exert inhibitory effects on free radical formation.

## 1. Introduction

Intensive treatment of diabetes leads to a reduction in plasma levels of glycosylated hemoglobin (HbA1c), which is associated with a significant decrease in the development and progression of vascular and neurologic complications [1, 2]. At present, measurement of HbA1c level is considered the gold standard for assessing long-term glycemic control and is regarded as a key therapeutic target for the prevention of diabetes-related complications [3]. Although HbA1c level is a measure of metabolic control and the effectiveness of therapeutic interventions directed to control hyperglycemia, it does not reveal any information on the extent and frequency of blood glucose excursions [3]. In this regard, it is important to note that recent studies show that glycemic instability may present additional risk to the development of complications over that predicted by the mean glucose value alone [4]. This being the case, HbA1c level may not always be the most clinically useful glycemic indicator of the risk for complications. Patients with similar mean glucose or HbA1c values can have different glycemic profiles, with differences both in the number and duration of glucose excursions [5]. It is therefore unknown whether two individuals with the same mean blood glucose (MBG) but extremes of glucose variability might have the same or different level of risk for complications.

Postprandial hyperglycemia may be a risk factor for cardiovascular disease in individuals with diabetes [6]. Endothelial dysfunction is one of the first stages, and one of the earliest markers, in the development of cardiovascular disease [7]. The possible deleterious roles of either postprandial hyperglycemia or glycemic variability were assessed via different markers of oxidative stress, and it was demonstrated that postprandial hyperglycemia independently induced endothelial dysfunction, through oxidative stress [8]. The fact that postprandial hyperglycemia induces oxidative stress is of particular significance. Recent studies demonstrated that hyperglycemia-induced overproduction of superoxide by the mitochondrial electron-transport chain and hyperglycemia-driven reactive oxygen species production enhanced four mechanisms of tissue damage via the polyol pathway, the hexosamine pathway, protein kinase C (PKC) activation, and formation of advanced glycation end-products (AGEs) [9].

With the apparent evidence that glycemic variability may be related to the pathogenesis of complications in diabetes [4] and in view of the need to reduce glycemic variability in order to achieve desired levels of control [10], it is important to have simple, clinically meaningful estimates of glycemic variability. MiniMed was the first commercial glucose sensor with FDA approval of the Continuous Glucose Monitoring System (CGMS) and includes a 3-day sensor and the necessary hardware to record the sensor current data and blood glucose measurements used for sensor calibration. Daily glycemic profiles can be recorded for 72 hours by CGMS, and intraday glycemic variability can be determined by the standard deviation (SD) around the mean glucose values. The recorded information is processed and analyzed retrospectively, which provides insights to improve insulin therapy [11].

This study applied continuous glucose monitoring technology to investigate the effect of high-dose insulin analog initiation therapy on glycemic variability and on formation of oxidative stress as determined from plasma and urine 8-iso prostaglandin F2*α* (8-iso PGF2*α*), plasma protein carbonyl, and nitrotyrosine levels. Plasma nitrite and nitrate levels were also determined to assess nitric oxide production.

## 2. Patients and Methods 

### 2.1. Determination of Patient Groups

The study group included 24 patients who were admitted to Antalya Research and Education Hospital, Endocrinology Clinic, with a diagnosis of type 2 diabetes mellitus (DM). HbA1c levels in all patients were above 10% despite ongoing therapy with sulphonylurea and metformin. Patients enrolled in the study were divided into three groups according to the given insulin treatment. Former treatment regimen was continued for the first day followed by substitution of sulphonylurea therapy with different insulin analogs. Group 1 (*N* = 8) received 0.4 U/kg/day lispro mix (50% insulin lispro protamine and 50% insulin lispro) subcutaneously (SC) in three equal doses plus 2000 mg/day oral metformin; Group 2 (*N* = 8) received 0.4 U/kg/day insulin aspart (30% insulin aspart and 70% protamine insulin aspart) SC in two equal doses plus 2000 mg/day oral metformin; Group 3 (*N* = 8) received 0.4 U/kg/day insulin glargine SC in one dose plus 2000 mg/day oral metformin. The given insulin treatments were in accordance with American Association of Clinical Endocrinologists (AACE) Diabetes Mellitus guidelines [12]. All patients gave written informed consent prior to entry. This study was approved by the Institutional Review Board of Akdeniz University School of Medicine and was performed in accordance with the Declaration of Helsinki.

### 2.2. Continuous Glucose Monitoring

All patients were equipped with CGMS (Medtronic MiniMed, USA) and were monitored for 72 consecutive hours after admission. A CGMS sensor was inserted into the subcutaneous abdominal fat tissue and calibrated according to the standard Medtronic MiniMed operating guidelines. During CGMS monitoring, blood glucose levels were checked via a glucometer (Accu-Check Go, Roche Co.) 4 times per day and the data was entered into the CGMS. After monitoring for 72 hours, the recorded data were downloaded into a personal computer for the analysis of the glucose profile. After downloading the recorded data, MBG levels and the SD around the mean glucose values, assessing glycemic variability, were analyzed from the data. 

### 2.3. Laboratory Measurements

Blood and urine samples were obtained from all patients at 24 and 72 hours. HbA1c levels were determined by Abbott ARCHITECT c16000 System (Abbott Diagnostic, Abbott Park, IL, USA) via immunoturbidimetric method. 

### 2.4. Measurement of Plasma Protein Carbonyl Levels

Plasma protein-bound carbonyls were measured via a protein carbonyl assay kit (Cat. no.1005020 Cayman Chemical, Ann Arbor, MI, USA). The utilized method was based on the covalent reaction of the carbonylated protein side chain with 2,4 dinitrophenylhydrazine (DNPH) and detection of the produced protein hydrazone at an absorbance of 370 nm. The results were calculated using the extinction coefficient of 22 mM^−1^ cm^−1^ for aliphatic hydrazones and were expressed as nmol/mL. As written in the instruction manual of the assay kit, typically human plasma has a protein carbonyl content of 35–280 nmol/mL. The intra- and interassay coefficients of variation (CV) for protein carbonyl measurements are 4.7% and 8.5%, respectively. 

### 2.5. Measurement of Plasma Nitrotyrosine Levels

Plasma nitrotyrosine content was measured via ELISA using a commercial kit (Cat. no.STA-305, Cell Biolabs, Inc. San Diego, CA, USA). Antigen captured by a solid phase monoclonal antibody (nitrated keyhole limpet hemocyanin raised in mouse) was detected with a biotin-labeled goat polyclonal antinitrotyrosine. A streptavidin peroxidase conjugate was then added to bind the biotinylated antibody. A TMB substrate was added and the yellow product was measured at 450 nm. A standard curve of absorbance values of known nitrotyrosine standards was plotted as a function of the logarithm of nitrotyrosine standard concentrations using the GraphPad Prism Software program for windows version 5,03. (GraphPad Software Inc). Nitrotyrosine concentrations in the samples were calculated from their corresponding absorbance values via the standard curve. The reported range of plasma nitrotyrosine levels in healthy human population determined via this assay kit is 20–148 nmol/L [13]. 

### 2.6. Measurement of Plasma Nitrite/Nitrate Levels

Plasma samples were transferred to an ultrafiltration unit and centrifuged through a 10-kDa molecular mass cut-off filter (Amicon, Millipore Corporation, Bedford, MA, USA) for 1 hr to remove proteins. Analyses were performed in duplicate via the Griess reaction using a colorimetric assay kit (Cayman Chemical, Cat. no.780001, Ann Arbor, MI, USA). The reported ranges of plasma nitrite/nitrate levels in healthy human population determined via this assay kit are 2–20 *μ*M [13]. The intra- and interassay coefficients of variation (CV) for nitrite/nitrate measurements are 2.7% and 3.4%, respectively. 

### 2.7. Measurement of Plasma-Free 8-iso Prostaglandin F2*α*

Plasma-free 8-iso PGF2*α* levels were determined by enzyme immunoassay (EIA) using 8-iso PGF2*α* EIA kit (Cayman Chemical, Cat. no.516351, Ann Arbor, MI, USA). Purification and extraction of plasma samples were performed before assay. Purification was done by 8-iso PGF2*α* affinity purification kit (Cayman Chemical, Cat. no.10368, Ann Arbor, MI, USA). The elution solution was evaporated to dryness by vacuum centrifugation via Savant DNA 120 speed vac concentrator (Thermo Scientific, IL, USA) and reconstituted with EIA buffer. As written in the instruction manual of the assay kit, plasma from human volunteers contains 40–100 pg/mL of 8-iso PGF2*α*. The interassay CV is 16.4% and 15.5% and intra-assay CV is 11.7% and 7.2% for 8-iso PGF2*α* measurements of 200 pg/mL and 12.8 pg/mL, respectively.

### 2.8. Measurement of Urine 8-iso Prostaglandin F2*α*

Urine-free 8-iso PGF2*α* levels were determined by enzyme immunoassay (EIA) using 8-iso PGF2*α* EIA kit (Cayman Chemical, Cat. no.516351, Ann Arbor, MI, USA). Isoprostane concentrations are expressed as nanograms per milligram of urine creatinine. As written in the instruction manual of the assay kit, normal human urinary levels of 8-iso PGF2*α* range from 10 to 50 ng/mmol creatinine. Urine creatinine levels were determined by colorimetric reaction (Jaffe reaction) of creatinine with alkaline picrate measured kinetically at 490 nm via commercial assay kit (Biolabo Reagents, Maizy, France). 

### 2.9. Statistical Analysis

Statistical analysis was performed by using SigmaStat statistical software version 2.0. Statistical analysis for each measurement is given in the result section.

## 3. Results

### 3.1. Patient Characteristics of Experimental Groups

Patient characteristics of experimental groups are given in Table 1.

### 3.2. CGMS Data of Experimental Groups

**Figure 1:**
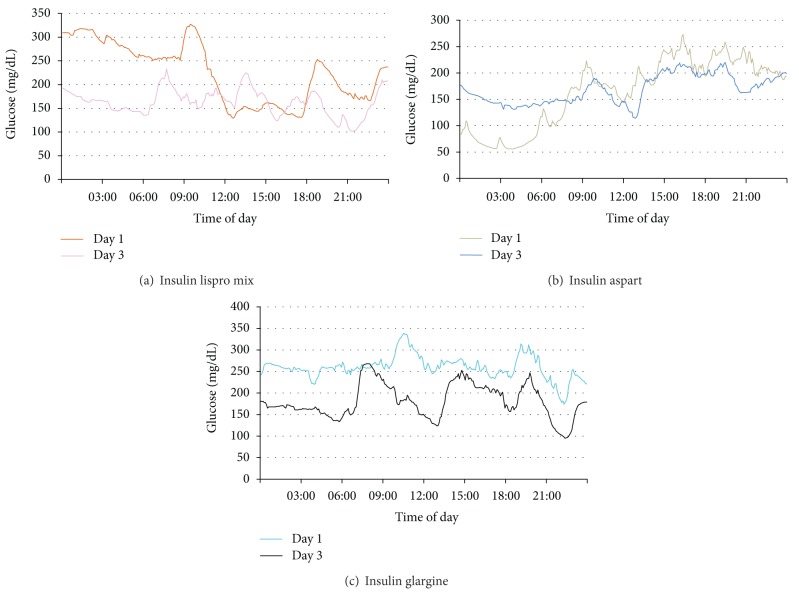
Representative micrographs of CGMS data from each experimental group. (a) Biphasic insulin lispro group, (b) biphasic insulin aspart group, and (c) insulin glargine group.

CGMS data of experimental groups are given in Table 2. A representative graph of CGMS results from each group is given in Figure 1. Mean blood glucose and SD around the mean glucose values after treatment with insulin analog plus metformin were significantly lower compared to those before treatment levels in all experimental groups. No significant difference was observed among different insulin analog treatments with respect to MBG and SD around the mean glucose values. Statistical analysis for MBG and SD levels was performed by two-way analysis of variance, and all pairwise multiple comparisons were done via Tukey test.

### 3.3. Plasma Protein Carbonyl Levels

**Figure 2:**
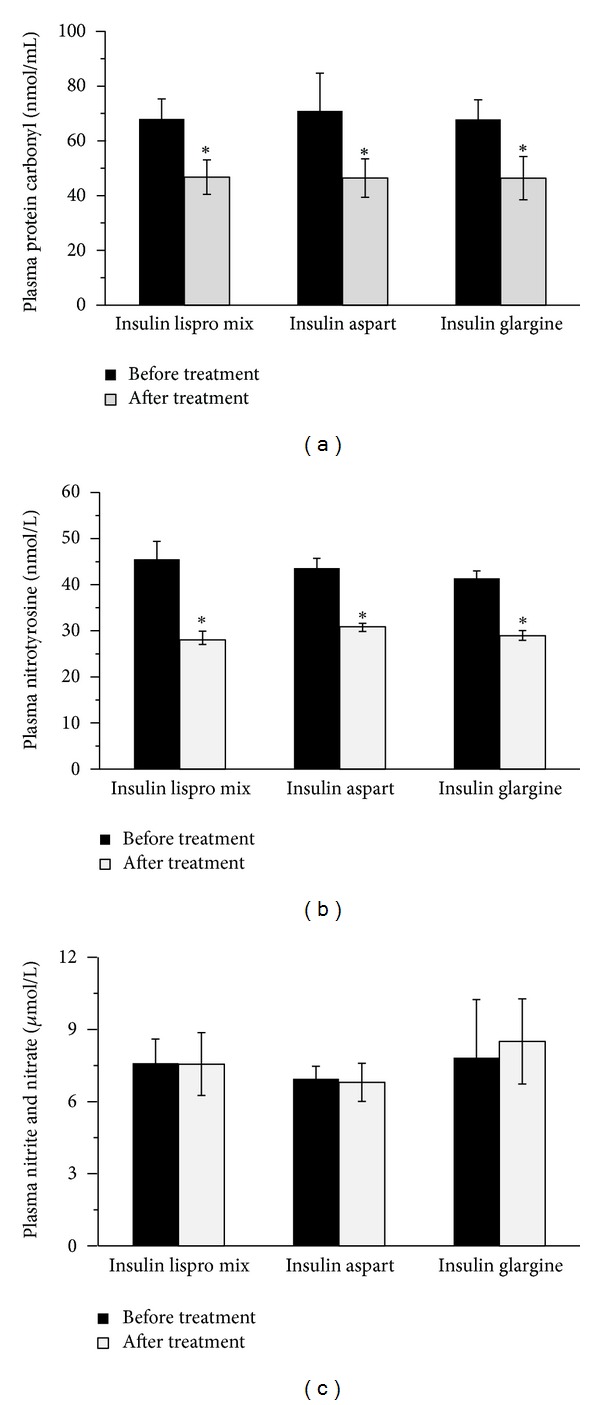
(a) Plasma protein carbonyl levels in experimental groups. *P < 0.001 compared to pretreatment. (b) Plasma nitrotyrosine levels in experimental groups. *P < 0.001 compared to pretreatment. (c) Plasma nitrite/nitrate levels in experimental groups.

Plasma protein carbonyl levels in treatment groups are given in Figure 2(a). Levels of plasma protein carbonyl (mean ± SD) were significantly (*P* < 0.001) decreased after treatment with insulin analog plus metformin (biphasic insulin lispro (*N* = 8), 46.78 ± 6.29; biphasic insulin aspart (*N* = 8), 46.42 ± 7.04; insulin glargine (*N* = 8), 46.39 ± 7.89 nmol/mL) compared to those before treatment levels in all experimental groups (biphasic insulin lispro (*N* = 8), 68.01 ± 7.34; biphasic insulin aspart (*N* = 8), 70.94 ± 13.79; insulin glargine (*N* = 8), 67.84 ± 7.20 nmol/mL). No significant difference was observed among different insulin analog treatments with respect to plasma protein carbonyl levels. Statistical analysis for plasma protein carbonyl levels was performed by two-way analysis of variance and all pairwise multiple comparisons were done via Tukey test. The correlation of plasma protein carbonyl levels with mean blood glucose values and SD around the mean glucose values following treatment with insulin analogs was evaluated by linear regression analysis. A significant correlation was observed between protein carbonyl levels and mean blood glucose values (*r* = 0.435, *P* = 0.033) (Figure 4(a)). Although levels of plasma protein carbonyl were significantly decreased after treatment with insulin analogs, no significant correlation was observed between glucose variability and plasma protein carbonyl levels (*r* = 0.055, *P* = 0.79). Likewise, changes in mean blood glucose from baseline to end point showed no significant correlation with changes in plasma protein carbonyl levels (*r* = 0.032, *P* = 0.882).

### 3.4. Plasma Nitrotyrosine Levels

Plasma nitrotyrosine levels are given in Figure 2(b). Plasma nitrotyrosine levels (mean ± SEM) were significantly (*P* < 0.001) decreased after treatment with insulin analog plus metformin (biphasic insulin lispro (*N* = 8), 28.01 ± 1.91; biphasic insulin aspart (*N* = 8), 30.86 ± 0.76; insulin glargine (*N* = 8), 28.93 ± 1.11 nmol/L) compared to those before treatment levels in all experimental groups (biphasic insulin lispro (*N* = 8), 45.11 ± 3.88; biphasic insulin aspart (*N* = 8), 43.61 ± 2.11; insulin glargine (*N* = 8), 41.36 ± 1.63 nmol/L). No significant difference was observed among different insulin analog treatments regarding plasma nitrotyrosine levels. Statistical analysis for plasma nitrotyrosine levels was performed by two-way analysis of variance and all pairwise multiple comparisons were done via Tukey test. The correlation of protein nitrotyrosine levels with mean blood glucose values and SD around the mean glucose values following treatment with insulin analogs was evaluated by linear regression analysis. A significant correlation was observed between nitrotyrosine levels and mean blood glucose values (*r* = 0.431, *P* = 0.035) (Figure 4(b)). Although levels of plasma nitrotyrosine were significantly decreased after treatment with insulin analogs, no significant correlation was observed between glucose variability and plasma nitrotyrosine levels (*r* = 0.10, *P* = 0.63). Likewise, changes in mean blood glucose from baseline to end point showed no significant correlation with changes in plasma nitrotyrosine levels (*r* = 0.066, *P* = 0.761). 

### 3.5. Plasma Nitrite/Nitrate Levels

Plasma nitrite/nitrate levels are given in Figure 2(c). No significant difference was observed in nitrite/nitrate levels (mean ± SEM) after treatment with insulin analog plus metformin (biphasic insulin lispro (*N* = 8), 7.56 ± 1.3; biphasic insulin aspart (*N* = 8), 6.8 ± 0.79; insulin glargine (*N* = 8), 8.5 ± 1.77 *μ*mol/L) compared to those before treatment levels in all experimental groups (biphasic insulin lispro (*N* = 8), 7.6 ± 0.99; biphasic insulin aspart (*N* = 8), 6.95 ± 0.52; insulin glargine (*N* = 8), 7.83 ± 2.4 *μ*mol/L). No significant difference was observed among different insulin analog treatments with regard to plasma nitrate/nitrite levels. Statistical analysis for plasma nitrite/nitrate levels was performed by two-way analysis of variance and all pairwise multiple comparisons were done via Tukey test.

### 3.6. Plasma-Free 8-iso Prostaglandin F2*α*

**Figure 3:**
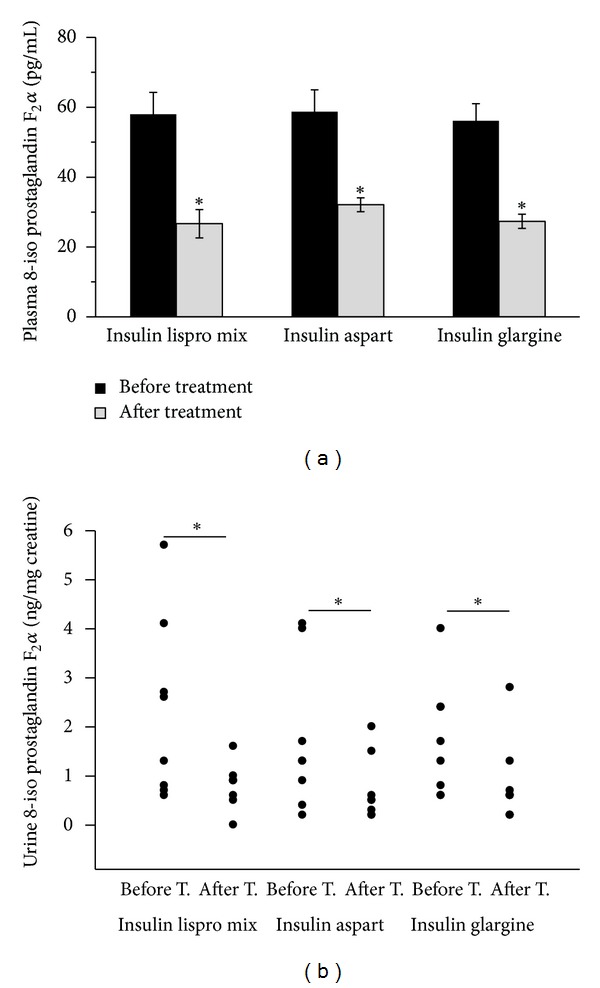
(a) Plasma-free 8-iso prostaglandin F2*α* levels in experimental groups. *P < 0.001 compared to pretreatment. (b) Urine 8-iso prostaglandin F2*α* levels in experimental groups.*P < 0.01 compared to pretreatment.

**Figure 4:**
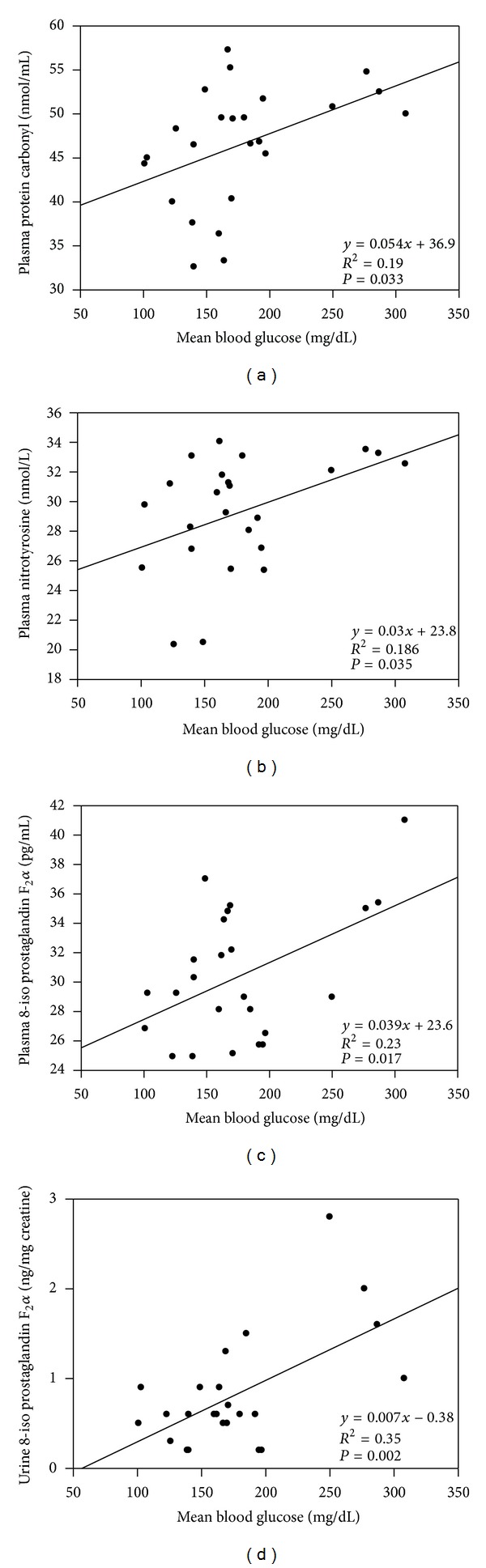
Scatter plots and correlation coefficients between mean blood glucose and oxidative stress markers following high-dose insulin analog therapy. (a) The correlation between mean blood glucose and plasma protein carbonyl levels, (b) the correlation between blood glucose and plasma nitrotyrosine levels, (c) the correlation between mean blood glucose and plasma-free 8-iso prostaglandin F2*α* levels, and (d) the correlation between mean blood glucose and urine 8-iso prostaglandin F2*α* levels.

Measured plasma-free 8-iso PGF2*α* levels are shown in Figure 3(a). Plasma-free 8-iso PGF2*α* levels (mean ± SEM) were significantly higher (*P* < 0.001) before treatment with insulin analogs plus metformin (biphasic insulin lispro (*N* = 8), 57.98 ± 6.3; biphasic insulin aspart (*N* = 8), 58.73 ± 6.23; insulin glargine (*N* = 8), 56.11 ± 4.89 pg/mL) compared to those after treatment levels (biphasic insulin lispro (*N* = 8), 26.67 ± 4.04; biphasic insulin aspart (*N* = 8), 32.10 ± 1.97; insulin glargine (*N* = 8), 27.36 ± 2.02 pg/mL). No significant difference was observed among different insulin analog treatments with respect to plasma-free 8-iso PGF2*α* levels. Statistical analysis for plasma-free 8-iso PGF2*α* was performed by two-way analysis of variance and all pairwise multiple comparisons were done via Tukey test. The correlation of plasma-free 8-iso PGF2*α* levels with mean blood glucose values and SD around the mean glucose values following treatment with insulin analogs was evaluated by linear regression analysis. A significant correlation was observed between plasma-free 8-iso PGF2*α* levels and mean blood glucose values (*r* = 0.481, *P* = 0.017) (Figure 4(c)). Although levels of plasma-free 8-iso PGF2*α* were significantly decreased after treatment with insulin analogs no significant correlation was observed between glucose variability and plasma-free 8-iso PGF2*α* levels (*r* = 0.06, *P* = 0.77). Likewise, changes in mean blood glucose from baseline to end point showed no significant correlation with changes in plasma-free 8-iso PGF2*α* levels (*r* = 0.045, *P* = 0.834).

### 3.7. Urine 8-iso Prostaglandin F2*α*

Urine 8-iso PGF2*α* levels are shown in Figure 3(b). Urine 8-iso PGF2*α* levels (mean ± SD) were significantly higher (*P* < 0.01) before treatment with insulin analogs plus metformin (biphasic insulin lispro (*N* = 8), 2.31 ± 1.84; biphasic insulin aspart (*N* = 8), 1.76 ± 1.51; insulin glargine (*N* = 8), 1.72 ± 1.17 ng/mg creatinine) compared to those after treatment levels (biphasic insulin lispro (*N* = 8), 0.81 ± 0.46; biphasic insulin aspart (*N* = 8), 0.71 ± 0.66; insulin glargine (*N* = 8), 0.87 ± 0.86 ng/mg creatinine). No significant difference was observed among different insulin analog treatments with respect to urine-free 8-iso PGF2*α* levels. Statistical analysis for urine 8-iso PGF2*α* levels was performed by two-way analysis of variance and all pairwise multiple comparisons were done via Tukey test. The correlation of urine-free 8-iso PGF2*α* levels with mean glucose values and SD around the mean blood glucose values following treatment with insulin analogs was evaluated by linear regression analysis. A significant correlation was observed between urine-free 8-iso PGF2*α* levels and mean blood glucose values (*r* = 0.593, *P* = 0.002) (Figure 4(d)). Although levels of urine-free 8-iso PGF2*α* were significantly decreased after treatment with insulin analogs, no significant correlation was observed between glucose variability and urine-free 8-iso PGF2*α* levels (*r* = 0.22, *P* = 0.3). Likewise, changes in mean blood glucose from baseline to end point showed no significant correlation with changes in urine-free 8-iso PGF2*α* levels (*r* = 0.165, *P* = 0.441). 

## 4. Discussion

The role of glycemic variability, assessed by using CGMS, in the formation of oxidative stress has been investigated in different studies. Mean amplitude of glycemic excursions and 24 h urinary excretion rates of 8-iso-PGF2*α* were calculated to determine glucose variability and oxidative stress, respectively. One study was performed in 21 type 2 diabetes patients and reported a strong correlation between glucose variability and oxidative stress [14]. Two other studies performed on 25 type 1 and 24 type 2 diabetic patients could not confirm a strong correlation between glucose variability and oxidative stress [15, 16]. To our knowledge, this is the first study to apply CGMS technology to investigate the effect of high-dose insulin analog initiation therapy on glycemic variability and on oxidative stress as determined from plasma and urine 8-iso PGF2*α*, plasma protein carbonyl, and nitrotyrosine levels.

Previous studies have reported increased levels of carbonyl groups in plasma proteins of type 2 diabetes mellitus subjects [17], while more recent studies have investigated the role of glycemic control in plasma protein oxidation. In one study, HbA1c levels were used as an index of glycemic control, and plasma carbonyl levels were measured in 17 patients with HbA1c >7% (poor glycemic control) and in 23 patients with HbA1c <7% (good glycemic control). A significant increase was reported in protein carbonyl levels in the poor glycemic control group [18]. In two similar studies, it was shown that type 2 diabetic patients with retinopathy and nephropathy had higher plasma levels of protein carbonyls as compared to type 2 diabetic patients without these complications [19, 20]. In a recent article, it was reported that elevated levels of carbonyl compounds correlated with insulin resistance in type 2 diabetes [21]. In support of previous studies, our data has shown that better glycemic control through initiation of high-dose insulin analog therapy resulted in decreased plasma protein carbonyl formation in type 2 diabetic patients. Although levels of plasma protein carbonyl were significantly decreased after treatment with insulin analogs, a significant correlation was not observed between SD around the mean glucose values and plasma protein carbonyl levels.

Previous studies have shown a significant increase in plasma nitrotyrosine levels in type 2 diabetic patients [22] and a significant correlation of nitrotyrosine values with plasma glucose concentrations (*r* = 0.38, *P* < 0.02) [23]. The role of hyperglycemia in postprandial nitrotyrosine generation was also investigated in 23 type 2 diabetic patients and 15 healthy subjects. Fasting nitrotyrosine was significantly increased in diabetic patients and was further increased during meal tests compared to controls. As compared with regular insulin, aspart administration significantly reduced the area under the curve of both glycemia and nitrotyrosine levels [24]. To our knowledge, this is the first study evaluating the effect of different insulin analogs on nitrotyrosine formation in type 2 diabetes. The observed decrease of plasma nitrotyrosine levels following high-dose insulin analog initiation therapy is in agreement with previous studies that show a significant correlation between nitrotyrosine levels and increased plasma glucose [23, 24]. The significant decrease of nitrotyrosine levels observed 48 hours after the initiation of insulin therapy supports the concept of increased transport of nitrated proteins across vascular endothelium [25]. Although we observed that plasma nitrotyrosine levels were significantly decreased after treatment with insulin analogs, we could not find a significant correlation between SD around the mean glucose values and plasma nitrotyrosine levels. 

Plasma and urine 8-iso PGF2*α* levels were significantly decreased following insulin analog initiation therapy. This observation is in accordance with the reported literature, which shows a link between glucose fluctuations and increased plasma and urine 8-iso PGF2*α* [14, 26]. In an observational study, 60 patients with type 2 diabetes were treated by oral hypoglycaemic agents alone, and 31 patients with type 2 diabetes were treated with insulin plus oral hypoglycaemic agents. Oxidative stress was estimated in these patients from 24 h urinary excretion rates of 8-iso PGF2*α*, and mean amplitude of glycemic excursions was estimated by CGMS. The 24 h excretion rate of 8-iso PGF2*α* was much higher (*P* < 0.001) in type 2 diabetic patients treated with oral hypoglycaemic agents alone than in type 2 diabetes group treated with insulin [27]. In a cross-sectional study recruiting type 2 diabetic patients, 17 patients were treated with basal bolus-insulin therapy, and 20 patients were treated with twice daily injection of premixed insulin analog therapy. No significant difference was observed in urinary 8-iso PGF2*α* levels in the two patient groups, and it was suggested that premixed insulin analog therapy was equivalent to basal bolus-insulin therapy in terms of glycemic fluctuations and oxidative stress [28]. A recent observational study was performed on 122 persons with type 2 diabetes, 61 were treated with oral hypoglycaemic agents alone, and 61 were treated with a combination of oral hypoglycaemic agents and insulin at either a low dose (<0.40 unit/kg/day) or high dose (≥0.40 unit/kg/day). The 24-h excretion rates of 8-iso PGF2*α* were much lower in patients receiving combination of oral hypoglycemic agents and insulin at a low dose [29]. A recent study also examined the relation between glycemic variability and oxidative stress in a cohort of type 2 diabetic patients treated with oral hypoglycemic agents. Twenty-four patients with type 2 diabetes underwent 48 hours of continuous glucose monitoring, and two consecutive 24-hour urine samples were collected for the determination of 8-iso PGF2*α* using high-performance liquid chromatography tandem mass spectrometry. Standard deviation and mean amplitude of glycemic excursions were calculated as markers of glycemic variability. Regression analysis showed no relevant relationship between glucose variability and 8-iso PGF2*α* excretions in patients enrolled in the study [16]. In our study, we also observed no significant correlation of urine-free 8-iso PGF2*α* levels with SD around the mean glucose values, evaluated by linear regression analysis. 

There are discrepancies among studies that have measured plasma nitrite/nitrate levels in type 2 diabetic patients. Some reports show increased levels of nitrite/nitrate in type 2 diabetic patients compared to healthy control subjects [30] while some studies show no difference among the two groups [31]. We observed no significant difference in nitrite/nitrate levels after treatment with insulin analogs plus metformin compared to those before treatment levels. Our observation is in agreement with a study which shows that metabolic control does not affect plasma levels of nitrate and nitrite in type 2 diabetic patients [32].

## 5. Conclusions

We have observed that treatment with insulin analog plus metformin resulted in a significant reduction in glycemic variability and oxidative stress as compared to oral hypoglycemic agents alone. The decrease in levels of oxidative stress markers, including plasma and urine 8-iso PGF2*α*, plasma protein carbonyl, and nitrotyrosine, following treatment with insulin analogs was significantly correlated with mean blood glucose levels. No significant correlation existed between glucose variability, determined by SD, and levels of plasma and urine oxidative stress markers. Likewise, changes in mean blood glucose from baseline to end point showed no significant correlation with changes in markers of oxidative stress. Data from this study suggests that treatment with insulin analogs, regardless of blood glucose changes, exerts inhibitory effects on free radical formation.

## Figures and Tables

**Table 1:** Patient characteristics of experimental groups.

Group	Age (years)	Gender (female/male)	HbA1c (%)
Insulin lispro mix (*n* = 8)	54.25 ± 16.10	4/4	11.94 ± 3.08
Insulin aspart (*n* = 8)	46.5 ± 9.7	4/4	11.91 ± 1.99
Insulin glargine (*n* = 8)	52.25 ± 6.48	3/5	11.48 ± 1.85

Data are mean ± SD. SD: standard deviation; HbA1c: hemoglobin A1c.

**Table 2:** CGMS data of experimental groups.

Group	Mean blood glucose (mg/dL) before treatment	SD (mg/dL) before treatment	Mean blood glucose (mg/dl) after treatment	SD (mg/dL) after treatment
Insulin lispro mix (*n* = 8)	227.00 ± 65.91	67.25 ± 28.53	183.25 ± 77.09^a^	39.38 ± 16.99^b^
Insulin aspart (*n* = 8)	187.75 ± 51.17	45.88 ± 17.06	165.88 ± 50.08^a^	32.50 ± 14.35^b^
Insulin glargine (*n* = 8)	231.63 ± 30.87	38.75 ± 10.59	182.75 ± 32.85^a^	32.25 ± 9.50^b^

Data are mean ± SD. SD: standard deviation; ^a^ *P* < 0.05 compared to mean blood glucose before treatment within the same group; ^b^ *P* < 0.01 compared to SD before treatment within the same group.
